# Specific Viruses Detected in Nigerian Children in Association with Acute Respiratory Disease

**DOI:** 10.1155/2011/690286

**Published:** 2011-10-11

**Authors:** Oluwabukola M. Akinloye, Esa Rönkkö, Carita Savolainen-Kopra, Thedi Ziegler, Bamidele A. Iwalokun, Mope A. Deji-Agboola, Afolabi Oluwadun, Merja Roivainen, Festus D. Adu, Tapani Hovi

**Affiliations:** ^1^Viral Infections Unit, Department of Vaccination and Immune Protection, National Institute for Health and Welfare (THL), Helsinki, Finland; ^2^Intestinal Viruses Unit, Department of Infectious Disease Surveillance and Control, National Institute for Health and Welfare (THL), Helsinki, Finland; ^3^Department of Medical Microbiology and Parasitology, Obafemi Awolowo College of Health Science, Olabisi Onabanjo University, Shagamu, Ogun State, Nigeria; ^4^Department of Virology, College of Medicine, University of Ibadan, Ibadan, Oyo State, Nigeria

## Abstract

Occurrence of different viruses in acute respiratory tract infections of Nigerian children was examined. Respiratory swabs were collected from 246 children referred to hospital clinics because of acute respiratory symptoms from February through May 2009. Validated real-time RT-PCR techniques revealed nucleic acids of at least one virus group in 189 specimens (77%). Human rhinoviruses and parainfluenza viruses were present each in one third of the children. Adenoviruses, enteroviruses, human metapneumovirus, human bocavirus, and influenza C virus were also relatively common. Possibly due to their seasonal occurrence, influenza A and B virus, and respiratory syncytial virus were detected rarely. We conclude that all major groups of respiratory tract viruses are causing illness in Nigerian children.

## 1. Introduction

Wide range of viruses is known to be associated with respiratory disease in humans. Adenoviruses, coronaviruses, human enteroviruses (HEV), human rhinoviruses (HRV), influenza viruses, parainfluenza viruses (PIV), and respiratory syncytial viruses (RSV) are well-known causes of acute respiratory tract infections (ARTI) in both industrialized and developing countries. Over the last decade, modern molecular techniques have led to the discovery of several previously unknown respiratory tract viruses, including human metapneumovirus (hMPV) [1], two new human coronavirus types [2, 3], human bocavirus (HBoV) [4], and two new human polyomaviruses [5, 6]. The significance of these novel viruses has been reviewed recently [7, 8].

It is widely accepted that common cold is almost always caused by viruses, most frequently by HRV [9], and viral infections are considered to contribute to the generation of complications of common cold, such as acute otitis media and sinusitis. Moreover, different viruses, including influenza viruses and RSV, are also frequently detected in samples obtained from patients with lower respiratory tract infection (LRTI), either alone or together with pathogenic bacteria. Several recent reports, including some from Africa, suggest viruses as potential etiologic agents in pneumonia in children [10–13], or exacerbations of asthma [14–16]. 

Several studies underscore the importance of respiratory tract viruses in Nigerian patients, but these studies were carried out before the introduction of modern molecular diagnostic techniques [14, 17–19]. The present study was designed to identify viral agents associated with respiratory infections among young children in Nigeria using modern, validated molecular techniques. We wanted to explore the presence of different virus groups, including some of the newer ones detected by only molecular techniques.

## 2. Materials and Methods

### 2.1. Patients and Specimens

The study was approved by the Ethical Committee of the Oyo State Ministry of Health. Participation of children in the study was voluntary and required informed consent from the parents. Inclusion criteria were recent onset of symptoms suggestive for respiratory tract infection, such as cough, coryza, repeated sneezing, and/or difficulty in breathing. Patients were recruited between February and May, 2009, and included hospitalized patients, children seen at emergency departments, and outpatient clinics at 3 different children's hospitals in Ibadan, Oyo state. Demographic and clinical information, including age, sex and clinical symptoms, was recorded during the medical visit by means of a structured questionnaire. 

A nasal swab sample was obtained from children by inserting a sterile nylon swab (Regular Flocked swab, Cat. No. 520CS01, Copan Diagnostics Inc., Murrieta, Calif USA) into the nostril to a depth of 2–4 cm, and retracting it with a rotating motion, in order to trap epithelial cells in the swab. With a second swab, a throat specimen was collected by rubbing the tonsils and the posterior wall of the pharynx. The 2 swabs were then placed in a vial containing 2 mL of RNAlater solution (RNA*later* Tissue Collection, Applied Biosystems, Espoo, Finland). The specimens were transported to the laboratory on the same day in an ice pack and stored at −70°C until further processing.

### 2.2. Detection of Viruses

Viral nucleic acids were extracted from 200 *μ*L of sample using a commercial kit (RNeasy Mini Kit, Qiagen, Hilden, Germany), and viral RNA was reverse transcribed into cDNA with random hexamer primers (Roche, Mannheim, Germany) and RevertAid reverse transcriptase enzyme (Fermentas, St. Leon-Rot, Germany) following the manufacturers' instructions. For the detection of influenza A and B viruses, RSV, PIV 1, 2, and 3, and cDNA were amplified in two separate real-time multiplex PCRs [20] with minor modifications. Other, slightly modified real-time PCRs were used for the detection of influenza A subtypes H1 and H3 (R. Fouchier, Rotterdam, the Netherlands, personal communication), influenza C (L. P. Nielsen, Copenhagen, Denmark, personal communication), bocavirus [21], metapneumovirus [22], and adenovirus [23]. Real-time RT-PCR for the detection of HRV was performed as described [24] and of enteroviruses (HEV) according to the method by Centers for Disease Control and Prevention, Atlanta, USA (W.A. Nix and D.R. Kilpatrick, personal communication). We know that the HEV test is not 100% specific but also reacts with some HRV strains (Savolainen-Kopra et al., unpublished results). Therefore, specimens yielding a positive result in the HEV test were divided into two groups, and a designated “true HEV” result was scored only for those HEV-positive specimens that were negative in the HRV test.

## 3. Results

More than half of the patients (132/246, 53.6%) were less than one year of age, and almost one half of the remaining children, whose age was recorded (40/83, 48.1%), were under two years of age (Table 1). Respiratory tract viruses were detected in 189 (77%) of the 246 children. The overall rate of virus positivity was similar in children less than 1 year of age (102/132, 77.3%), between 1 and 2 years (32/40, 80.0%), and above 2 years of age (33/43, 76.7%) and slightly lower among the 13% of children whose age was not recorded (22/31, 71.0%). The most frequently detected virus groups, both found in about one third of the children each, were HRV and PIV, with type 3 of PIV being the most prevalent serotype in the latter group (Table 1). Adenoviruses, influenza virus C, hMPV, and HBoV were also found in considerable numbers. Influenza virus types A and B and RSV were detected less frequently. Seven specimens tested positive for HEV and negative for HRV (“true HEV”), whereas 16 other additional specimens yielded a positive result by the HEV and the HRV test. While the overall proportion of virus-positive specimens was similar in children aged under or over two years, as well as in the group with unrecorded age, all adenovirus, influenza virus A, RSV, and all but one HBoV and “true HEV” detections were in the youngest age group (Table 1).

Altogether 224 virus findings were obtained from the 246 children, if only those HEV-positive results were accounted where the same specimen was negative in the HRV test (“true HEV”). The number of findings was 240 if all positive test results for HEV were included. Twenty-nine specimens contained two different viruses and another two specimens three different viruses. No obvious pattern could be seen in the mutual associations of two or three viruses (Table 2). However, there were more multiple infections in children older than 2 years than in the younger than 2 years group (33% versus 9% of children, *P* = 0.0001 (2-sample *t*-test for equality of proportions), data not shown.

## 4. Discussion

This study revealed that all major respiratory virus groups tested for can be detected in Nigerian children with respiratory tract infection. All viruses investigated, including the more recently discovered HMPV and HBoV, were present in at least some of the specimens. This wide variety is somewhat surprising because the specimens have been collected only during a four-month period, mostly in the dry season of the year. It is known from the previous studies that circulation of some viruses is at a low level during the dry season [25–27]. Seventy-seven percent of the children harboured at least one viral pathogen in their samples. This figure is relatively high taking in account that we did not have the possibility to test the specimens for human coronaviruses, and comparable to [28] or higher than in some recent similar studies performed elsewhere [29–31]. Human rhinoviruses and PIV were the two most frequently detected virus groups. Simultaneous infections with two or even three viruses were also found, similar to observations by others in comparable studies [9, 25]. 

In individual cases, a demonstration of viral nucleic acids in a nasal or throat swab does not necessarily prove an etiological role of this pathogen in the concurrent disease as positive test results have also been obtained from a proportion of healthy individuals [32]. Etiological diagnosis of LRTI is especially difficult without invasive procedures, but when searched for, for example, rhinovirus has been found in alveolar lavage specimens from pneumonia patients [33] establishing the role of HRV as a significant respiratory pathogen beyond being the major causative agent of common cold [15].

Previous studies on respiratory tract viruses in Nigeria have not tested for the presence of rhinoviruses. In our study, HRV accounted for 39% of the virus findings in the study population reiterating the observations done elsewhere [9, 29, 30, 34]. Our test for the related HEV revealed a positive result in about 10% of the specimens, but two thirds of these specimens also tested positive for HRV. Because of the well-established cross-reactions [24], these double-reactive specimens were recorded as HRV, and only those 7 HEV-positive specimens that were HRV negative were scored as “true HEV positives.” However, we cannot exclude concomitant infection with both HEV and HRV in these double-reactive cases.

A high incidence of parainfluenza viruses was found in our study population with PIV3 being the most frequent of the three serotypes tested for. Interestingly, PIV3 has also been one major agent in some previous studies done in Nigeria [14, 17] and one in Cambodia [29]. Respiratory syncytial virus was a rare finding in our study, likely due to the seasonal occurrence of this virus. Our specimens were collected in February-May which has been a low season for RSV in Nigeria in previous studies [26, 27].

Little was known about the epidemiology of influenza viruses in West Africa until very recently. A report from Senegal [25] published, while this manuscript was in preparation, showed a September-October peak of influenza A and RSV. Hence, seasonality might have again been contributing to the paucity of influenza virus A and B findings among our patients. The two influenza A viruses further characterized were of the H3N2 subtype, that is, the same subtype that was predominant during the 2008-2009 influenza season (http://apps.who.int/globalatlas/dataQuery/default.asp). In contrast, influenza virus type C was found surprisingly often. With an incidence of almost 5%, influenza C was as common as adenoviruses in this study.

Double or triple infections were detected in 16% of the virus-positive children, which is comparable to that observed by others [31, 34]. We do not have any explanation for the observed relatively higher frequency in the older children. One could speculate about increasing number of contacts by age in the community, but we did not collect information enabling further analysis of this possibility.

In conclusion, the findings of this study emphasize the presence of all major groups of viruses in association with respiratory illnesses in young children of West Africa. Rhinoviruses and parainfluenza viruses were the most prevalent virus groups while influenza A and B viruses, as well RSV were rarely detected, possibly due to low season of these viruses during the time of sample collection.

## Figures and Tables

**Table 1:** Distribution of viral pathogens according to the age of the patients.

Age (years)	*N*	Virus Neg	Adeno	HBoV	HEV* total	HEV* HRV-	HMPV	HRV	Infl. A	Infl. B	Infl. C	PIV1	PIV2	PIV3	RSV
<1	132	30	8	4	12	5	2	47	1	0	4	6	6	33	1
1-2	40	8	4	1	4	1	0	17	2	1	4	3	4	8	0
2-3	24	4	0	0	2	0	2	6	0	0	2	4	8	1	0
3-4	7	1	0	0	0	0	1	2	0	1	0	1	1	2	0
4-5	9	4	0	0	1	0	1	2	0	0	1	0	0	1	0
>5	3	1	0	0	0	0	0	1	0	0	0	0	0	0	0
no age	31	9	0	1	4	1	1	12	0	0	1	1	2	5	0

Total	246	57	12	6	23	7	7	87	3	2	12	16	21	50	1

*Our HEV test can react with some HRV strains. “Total” shows the number of all specimens testing positive in the HEV assay, “HRV-” those where the HRV test was negative (“true HEV positives”).

**Table 2:** Coappearance of viral pathogens in patients with respiratory tract infection.

			Number of specimens with indicated virus detected											
Virus			Double infection with indicated virus											
	Total	Alone	Adenovirus	Human bocavirus	Human enterovirus	Human metapneumovirus	Human rhinovirus	Influenza virus A	Influenza virus B	Influenza virus C	Parainfluenza virus 1	Parainfluenza virus 2	Parainfluenza virus 3	Respiratory syncytial

*Adenovirus*	12	7	X	0	0	0	2	0	0	2	0	0	1	0
*Humanbocavirus*	6	1	0	X	0	0	2, a	0	0	0	0	0	1, a	1
Human enterovirus*	7	5	0	0	X	0	0	0	0	1	0	0	1	0
*Human metapneumovirus*	7	4	0	0	0	X	2	0	0	0	0	0	1	0
*Human rhinovirus*	87	68	2	3, a	0	2	X	0	0	1	b	3b	7, a	0
*Influenza virus A*	3	2	0	0	0	0	0	X	0	0	0	1	0	0
*Influenza virus B*	2	1	0	0	0	0	0	0	X	0	0	0	1	0
*Influenza virus C*	12	8	2	0	1	0	1	0	0	X	0	0	0	0
*Parainfluenza virus 1*	16	14	0	0	0	0	B	0	0	0	X	1, b	0	0
*Parainfluenza virus 2*	21	15	0	0	0	0	3, b	0	0	0	1, b	X	1	0
*Parainfluenza virus 3*	50	36	1	1, a	1	1	7, a	0	1	0	0	1	X	0
*Respiratorysyncytial virus*	1	0	0	1	0	0	0	0	0	0	0	0	0	X

Two triple infections, one with bocavirus, human rhinovirus, and parainfluenza virus 3 (a) and the other with human rhinovirus, parainfluenza virus 1 and parainfluenza virus 2 (b) were detected.

*Only the “true HEV” positive specimens are included.
